# Ambulatory Blood Pressure Monitoring During 52 Hours in Patients With Chronic Kidney Disease and Haemodialysis Treatment—An Exploratory Pilot Study

**DOI:** 10.1111/jorc.70009

**Published:** 2025-02-05

**Authors:** Jenny Stenberg, Oskar Sandberg, Kerstin Marttala, Maria K. Svensson

**Affiliations:** ^1^ Department of Medical Sciences, Renal Medicine Uppsala University Hospital Uppsala Sweden; ^2^ Uppsala Clinical Research Centre Uppsala Sweden

**Keywords:** ambulatory blood pressure, chronic kidney disease, dialytic interval, haemodialysis

## Abstract

**Background:**

Hypertension in patients with haemodialysis is mainly characterised by high pre‐dialysis blood pressure (BP) due to body fluid retention before dialysis, and the BP tends to decrease after dialysis due to removal of water during dialysis. Intervals between haemodialysis treatments vary and a long inter‐dialytic interval dialysis is associated with increased mortality.

**Objective:**

To examine variations in BP; how ambulatory BP vary during a dialysis treatment performed after the long inter‐dialytic interval, that is, the first treatment of the week, compared to dialysis after a short inter‐dialytic interval, and in the interval between the two dialysis sessions.

**Design:**

Exploratory non‐interventional observational.

**Participants:**

Eleven patients with chronic kidney disease Stage 5 treated with haemodialysis were enroled. The mean age was 69 years (range 48–87) and mean dialysis duration 65 months (range 4–128).

**Measurements:**

Ambulatory BP was recorded for 52 h including two haemodialysis treatments and one inter‐dialytic interval. For statistical analyses the Wilcoxon signed ranks test was used.

**Results:**

Significant differences in systolic and diastolic BPs were observed between dialyses after long inter‐dialytic interval and short inter‐dialytic interval, respectively (systolic 122 mmHg vs. 114 mmHg, *p* = 0.012 and diastolic 62 mmHg vs. 61 mmHg, *p* = 0.036). In addition, the BP declined during the first 90 min during dialysis in both settings.

**Conclusions:**

Measuring ambulatory BP for 52 h in patient with chronic kidney disease and haemodialysis is feasible and show that both systolic and diastolic BP differ between dialysis treatments following inter‐dialytic intervals of diverse length. These findings should be replicated in larger studies.

## 1 Introduction

Patients with chronic kidney disease (CKD), Stage 5 and haemodialysis treatment have nearly a 20% annual mortality rate, with cardiovascular death being the primary cause (Thompson et al. 2015; Bello et al. 2022). The prevalence of hypertension in these patients is nearly 90% and associated with increased cardiovascular morbidity and mortality (Navaneethan et al. 2017; Burnier and Damianaki 2023). Hypertension in patients with haemodialysis is mainly characterised by high pre‐dialysis blood pressure (BP) due to body fluid retention before dialysis, and the BP tends to decrease after dialysis due to removal of water during dialysis (Sarafidis et al. 2017; Kim et al. 2023).

## 2 Literature Review

BP and volume control are important in dialysis care and have impacts on patient symptoms, quality of life, and cardiovascular complications and frequent BP measurements are also necessary to ensure patient safety during haemodialysis treatments (Flythe et al. 2020). Patients with intradialytic hypertension, or increases in BP during haemodialysis, have been shown to have the highest risk for increased cardiovascular morbidity and mortality (Van Buren and Inrig 2016). However, the linear relationship between increasing systolic BP and cardiovascular mortality in the general population does not exist in haemodialysis patients. Instead, a U‐shaped curve best describes the relationship between single BP measurements, that is, high and low systolic BP and pulse pressure at the haemodialysis clinic, and mortality (Jhee et al. 2018). In addition, BP should normally drop at night, if not, these patients are defined as “non‐dippers”. The pattern is linked to hypertension, and is seen in a large proportion of patients with CKD Stage 5 (Burnier and Damianaki 2023).

Intervals between haemodialysis treatments vary, and a long inter‐dialytic interval (LIDI) dialysis is associated with increased mortality. It has been shown that, in patients treated with in‐centre haemodialysis three or fewer times per week, cardiovascular events—which account for 40% of deaths—are significantly more likely to occur on the first dialysis session of the week (Foley et al. 2011; Krishnasamy et al. 2013). However, it remains uncertain whether BP variability is a modifiable risk factor or a marker of underlying pathology (e.g., volume shifts, arterial stiffness) (Flythe et al. 2020).

Home and ambulatory blood pressure (AMBP) measurements from the inter‐dialytic period have been shown to be better predictors of mortality than individual pre or post haemodialysis BP measurements at the haemodialysis clinic (Khan et al. 2016; Sarafidis et al. 2017; Mayer et al. 2018; Georgianos et al. 2022). AMBP usually involves repeated BP measurements over at least 24 h. In treatment guidelines, the threshold for ambulatory hypertension target value for AMBP is 130/80 mmHg (Mancia et al. 2023). Given the BP variability attributed to inter‐dialytic fluid overload, 44‐h ABPM has been suggested to better delineate cardiovascular morbidity in patients treated with haemodialysis (Agarwal et al. 2014). However, since dialysis is usually administered three times a week, even a 44‐h observation period may be insufficient to capture the variations in BP across different intervals.

There is a need to improve clinical practice by refining and individualising BP monitoring strategies, but to our knowledge no previous study have extended the observation time to include both dialysis sessions succeeding a short and a long inter‐dialytic interval and the time in‐between. The objective of this study was to investigate how intradialytic AMBP differs between two different dialysis sessions after different inter‐dialytic intervals and during the inter‐dialytic interval in patients with CKD treated with haemodialysis. The intention was also to acquire knowledge about how the measuring equipment for AMBP can be used in connection with dialysis treatment and how an extended examination time (52 h) is tolerated by patients.

## 3 Materials and Methods

In this exploratory non‐interventional observational study 11 patients with CKD Stage 5 treated with haemodialysis at the department of Nephrology, Uppsala University Hospital were enroled. Patients were purposively selected, and recruiting was primarily based on recommendations from the staff at the clinic and the patients presumed motivation to participate in a research study. The study complied with the Declaration of Helsinki and was approved by the Regional Ethics Committee in Uppsala (dnr 2021‐05658‐01). Written informed consent was obtained from all participants. Descriptive clinical characteristics were collected from electronic health records.

Before dialysis treatment, patients received the Labtech EC‐3H device applied together with a BP cuff to the non‐fistula arm for BP recording and AMBPs were recorded up to 52 h. The choice of device was based on availability at the clinic. During the day, BP was measured automatically every 30 min and at night‐time every 120 min. During the dialysis treatment, the cuff was temporarily removed for the patients with dialysis fistula and BP values were measured at the same intervals via the dialysis machine and noted on a printed template. After the 52 h, or the end of the second dialysis treatment, the equipment was removed and recorded measurements were transferred from the Labtech Cardioscopy software into Microsoft Excel manually by reading PDF files from Labtech Cardioscopy. If the equipment was not able to perform a measurement, this was reported by the software as a “false” value, and those were sorted out of the data set. The first and last BPs during dialysis treatment in the study were the first and last BPs during dialysis treatment with successful BP measurement.

Variations in BP during dialysis treatments after the LIDI and after a short inter‐dialytic interval (SIDI), as well as the first BP and last BP recording during these two dialysis treatments were compared. The inter‐dialytic period was a maximum of 44 h, and this interval was divided into two equal parts of a maximum of 22 h each (the first and second inter‐dialytic period), which were then compared. To enable the investigation of differences in AMBP between different inter‐dialytic intervals the average BPs for the pre‐defined intervals were calculated for each individual patient, using all values registered during the interval. However, for the comparison of BP pre and post HD, single BP recordings were analysed.

Due to the low number of study participants, and to reduce the risk for false positive findings, a non‐parametric statistical method, the Wilcoxon signed rank test was used to analyse differences in BP during LIDI and SIDI, for the first day, night or day compared to the second and first and last BPs during dialysis. *p*‐Values < 0.05 were considered significant. Data analyses were performed using IBM SPSS Statistics Version 28.0.1.

## 4 Results

Eleven patients were asked and consented to participate in the study. Clinical characteristics of the study participants are shown in Table 1. Most patients were men (72%), and chronic hypertensive nephropathy was the most common underlying cause of CKD. Three patients had dialysis intervals other than three times per week. Two of the patients had no need for ultrafiltration during dialysis. The prescribed dialysis treatment times varied between 4 and 5 h. The length of the SIDI was 1–3 days and the LIDI was 2–3 days. The time between the two dialysis treatments with AMBP was 43–44 h. All participants, except one, tolerated having the equipment on for the planned period.

**Table 1:** Clinical characteristics of study participants.

Variables		(*n* = 11)
Age (years)		69 (48–87)
Sex (women/men) *n* (%)		3 (27)/8 (73)
Target weight (kg)		74 (52–110)
Ultrafiltration rate (UFR; mL/h)		500 (0–900)
Kt/V		1.78 (1.32–2.51)
Urea reduction rate (URR; %)		78 (70–88)
Time on dialysis (months)		65 (4–128)
Access (AV‐fistula/CDC) *n* (%)		6 (54)/5 (46)
Diabetes *n* (%)		3 (27)
Cardiovascular disease *n* (%)		6 (55)
Antihypertensive medication *n* (%)		9 (82)
Dialysis frequency *n* (%)		
	Two times per week	2 (18)
	Three times per week	8 (73)
	Four times per week	1 (9)
Underlying cause of chronic kidney disease (CKD) *n* (%)		
	Chronic hypertensive nephropathy	4 (36)
	Systemic vasculitis	1 (9)
	Crescentic glomerulonephritis	1 (9)
	Diabetic kidney disease	2 (18)
	Anti‐glomerular basement membrane disease	1 (9)
	Renal cell carcinoma	1 (9)
	Adult polycystic kidney disease	1 (9)

*Note:* Data are means and range (minimum‐max) or numbers (*n*) and proportions (%).

Abbreviations: AV‐fistula: arterio‐venous fistula; CDC, central dialysis catheter.

**Figure 1:**
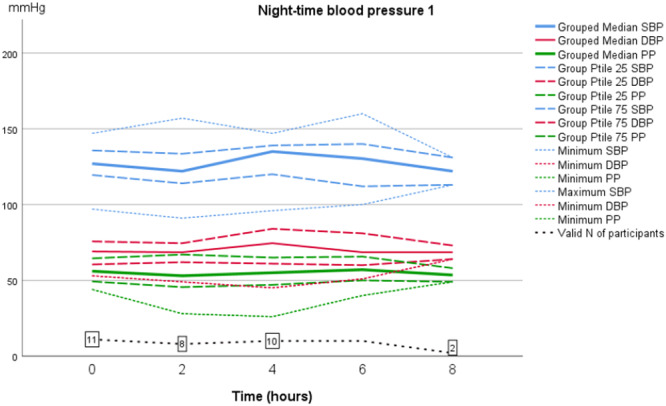
Night‐time inter‐dialytic ambulatory systolic and diastolic blood pressure measurements during the first inter‐dialytic period. Data are median, 25th–75th percentiles and number of valid observations (*N*).

**Figure 2:**
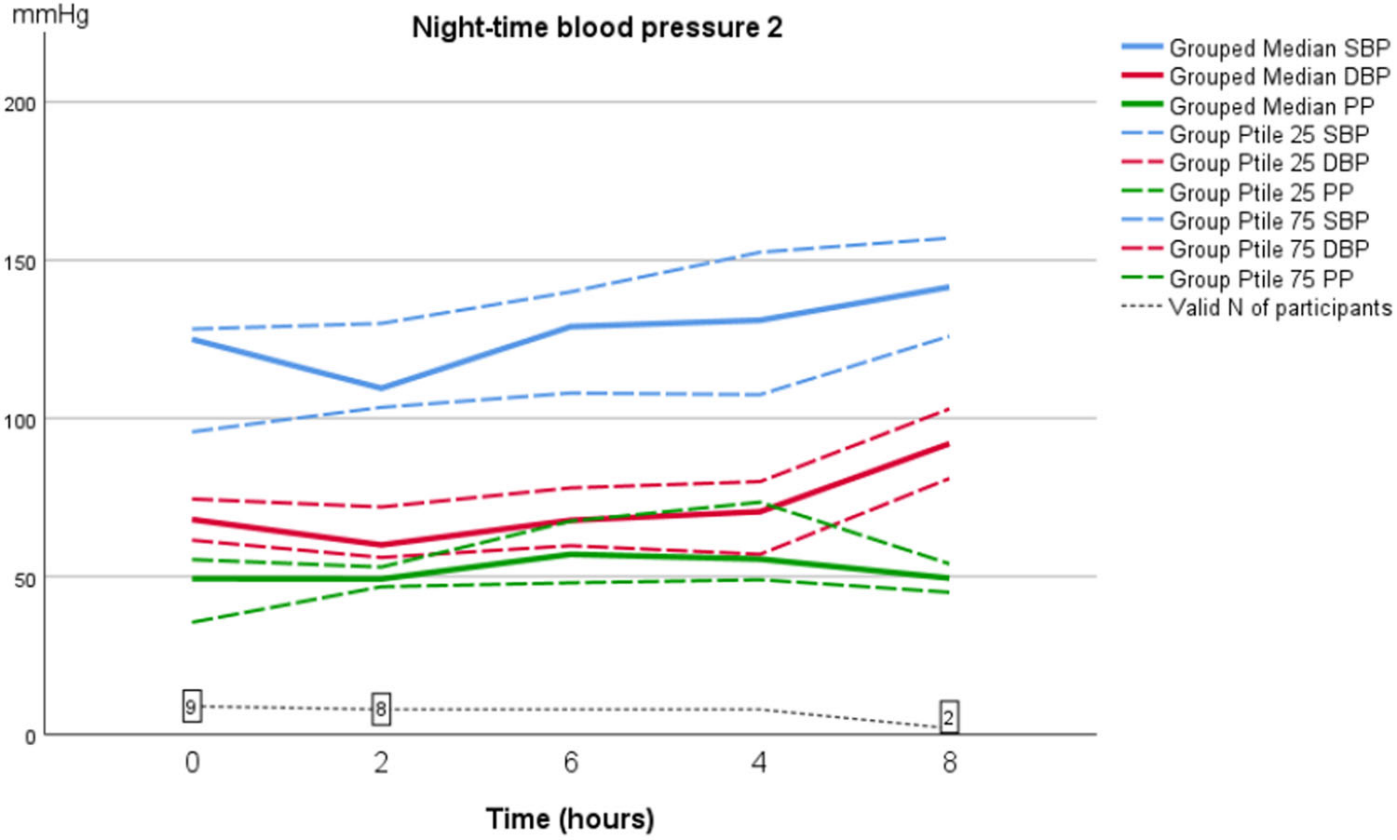
Night‐time inter‐dialytic ambulatory systolic and diastolic blood pressure measurements during the second inter‐dialytic period. Data are median, 25th–75th percentiles and number of valid observations (*N*).

**Figure 3:**
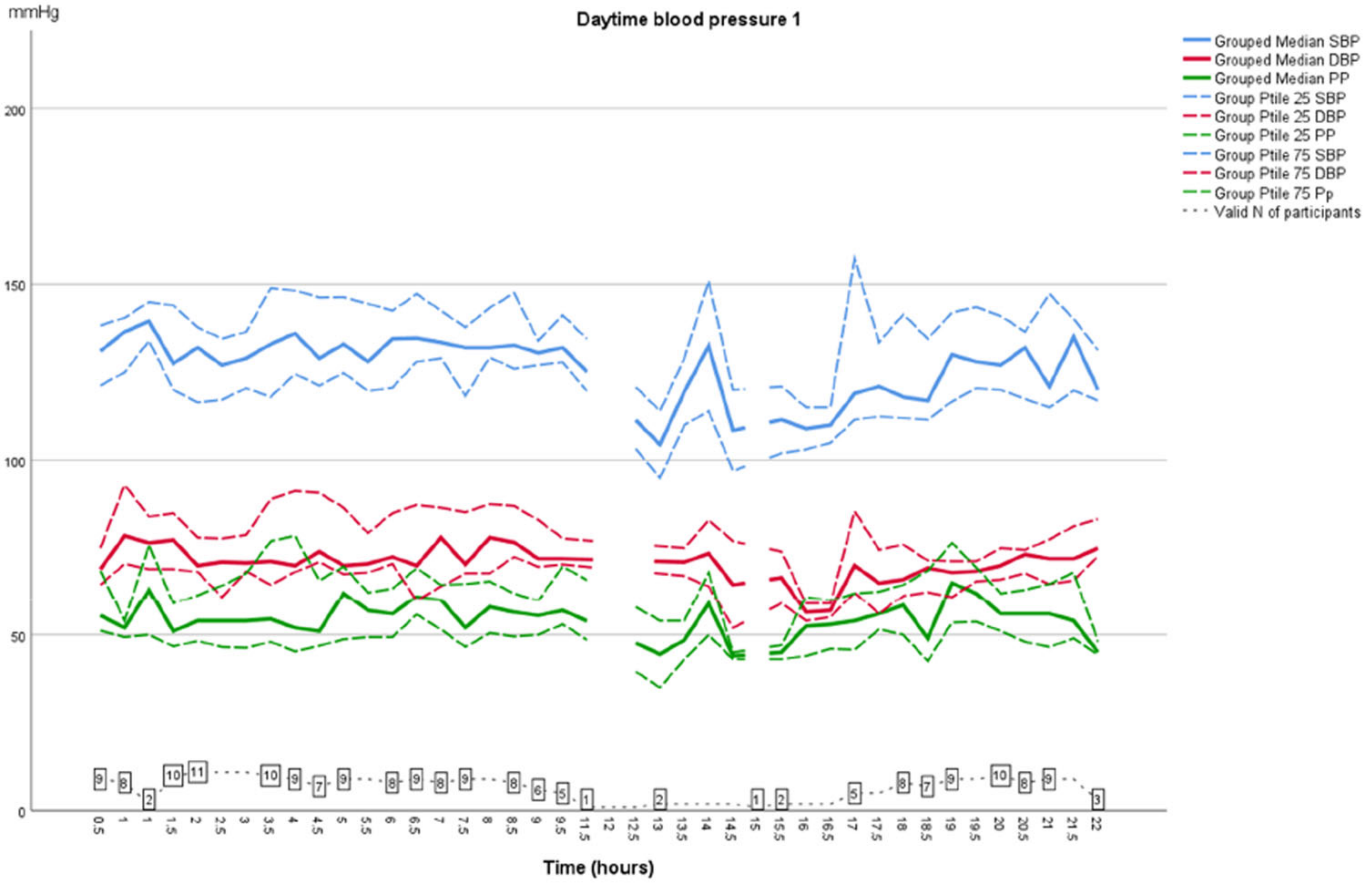
Daytime inter‐dialytic ambulatory systolic and diastolic blood pressure measurements during the first inter‐dialytic day. Data are median, 25th–75th percentiles (Ptile) and number of valid observations (*N*).

**Figure 4:**
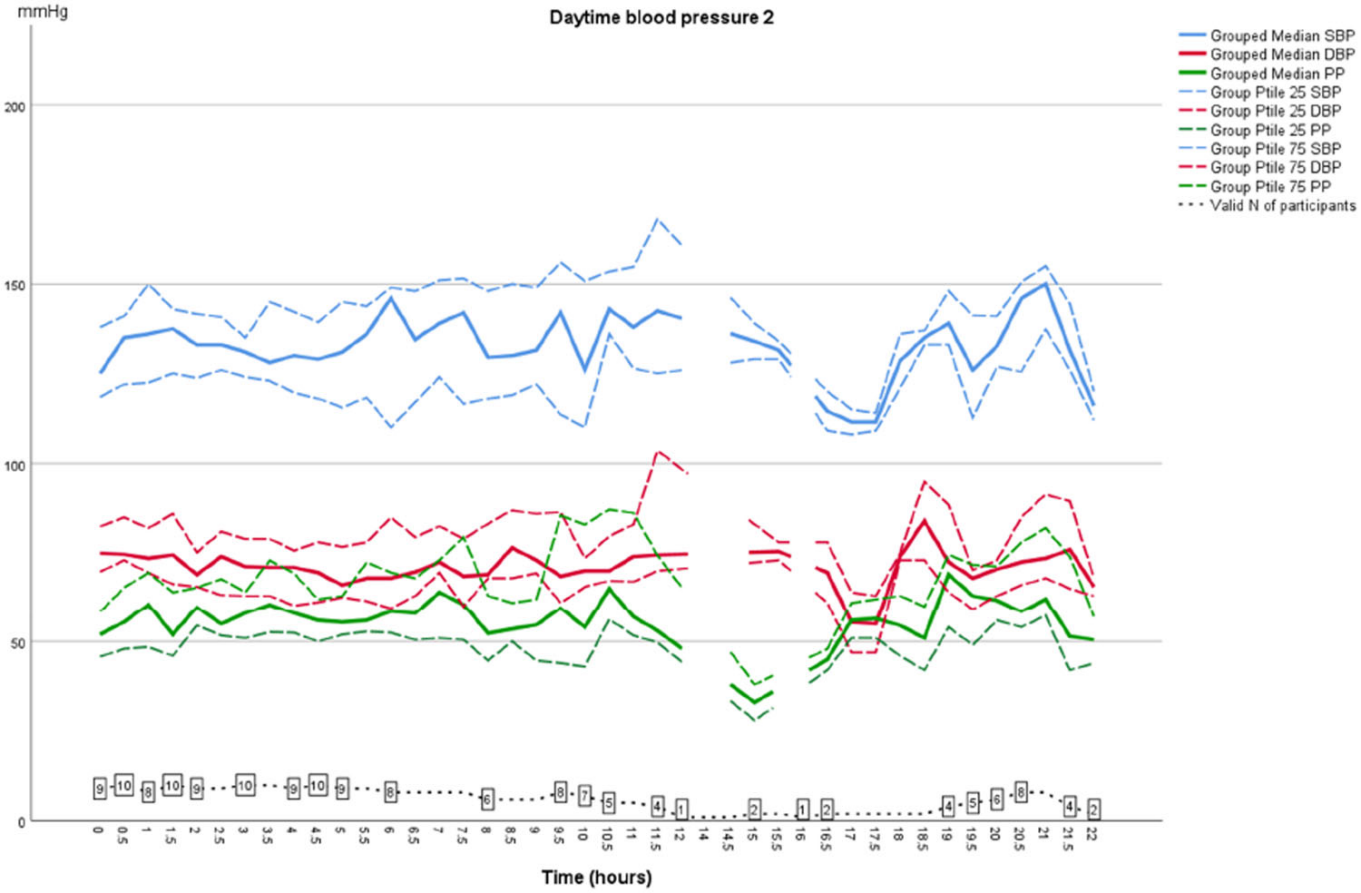
Daytime inter‐dialytic ambulatory systolic and diastolic blood pressure measurements during the second inter‐dialytic day. Data are median, 25th–75th percentiles and number of valid observations (*N*).

As shown in Table 2, during the first inter‐dialytic period the AMBP dropped at night‐time; median SBP dropped from 130 to 116 mmHg (14 mmHg; *p* = 0.594) and median DBP from 71 to 66 mmHg (5 mmHg; *p* = 0.314). However, during the second inter‐dialytic night BP did not drop, instead an increase in both SBP and DBP was observed at the fourth and fifth measurements. This difference however did not reach statistical significance (Figures 1 and 2, Table 2). At daytime no difference in AMBP was found between the first and second inter‐dialytic period (Figures 3 and 4). In addition, no change was observed in pulse pressure (data not shown).

**Table 2:** Differences in median ambulatory blood pressure between the first and second day of the inter‐dialytic interval.

Variable	Day 1	Day 2	*p*‐Value
Daytime SBP (mmHg)	130 (121–141)	132 (121–148)	0.386
Night‐time SBP (mmHg)	116 (111–137)	132 (120–138)	0.401
Daytime DBP (mmHg)	71 (67–81)	70 (68–78)	0.508
Night‐time DBP (mmHg)	66 (63–77)	70 (61–81)	0.889

*Note:* Variables presented as median and interquartile range (25th‐75th percentiles).

Abbreviations: DBP, diastolic blood pressure; SBP, systolic blood pressure.

After the LIDI mean AMBPs during dialysis, i.e. intradialytic BP, both systolic and diastolic, were significantly higher compared to after a SIDI (systolic BP 122 mmHg vs. 114 mmHg, *p* = 0.012; diastolic BP 62 mmHg vs. 61 mmHg, *p* = 0.036), Table 3.

**Table 3:** Comparisons of blood pressure measurements after the LIDI and a SIDI.

Variable	After LIDI	After SIDI	*p*‐Value
Intradialytic syst. AMBP (mmHg)	122 (115–136)	114 (104–125)	0.012*
Intradialytic diast. AMBP (mmHg)	62 (61–81)	61 (52–78)	0.036*
Pre‐HD SBP (mmHg)	131 (123–142)	122 (108–138)	0.207
Post‐HD SBP (mmHg)	128 (116–136)	114 (101–130)	0.068
Pre‐HD DBP (mmHg)	76 (71–92)	69 (54–82)	0.093
Post‐HD DBP (mmHg)	68 (61–77)	58 (55–77)	0.141

*Note:* Variables presented as median and interquartile range (25th–75th percentiles).

Abbreviations: AMBP, average of ambulatory blood pressure for a pre‐defined interval; DBP, single diastolic blood pressure; HD, haemodialysis; LIDI, dialysis treatment after a long inter‐dialytic interval; SBP, single systolic blood pressure measurements; SIDI, dialysis treatment after a long inter‐dialytic interval.

* *p* < 0.05.

**Figure 5:**
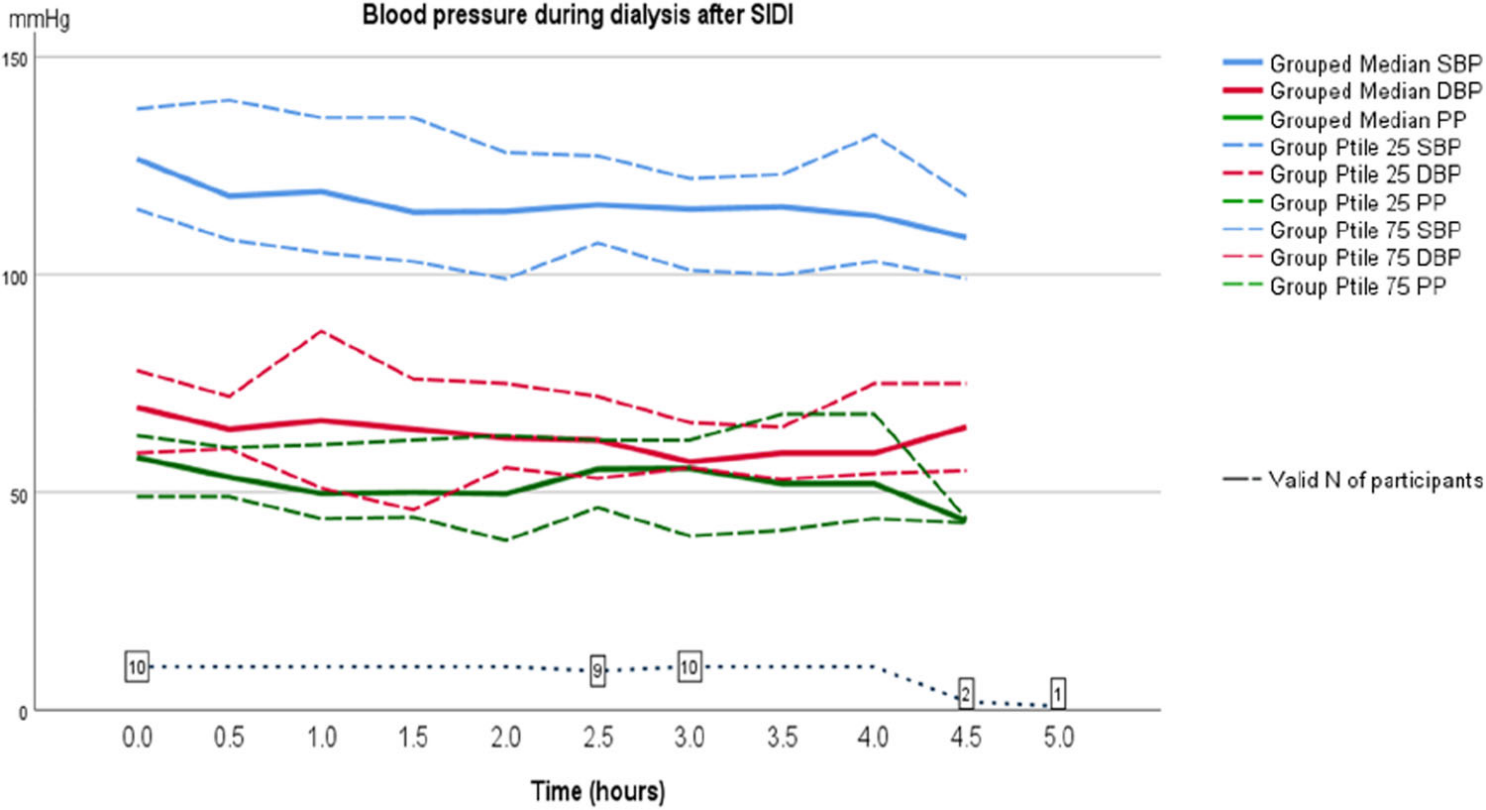
Ambulatory systolic and diastolic blood pressure (AMBP) measurements during the dialysis session after a short inter‐dialytic interval (SIDI). Data are median, 25th–75th percentiles and number of valid observations (*N*).

**Figure 6:**
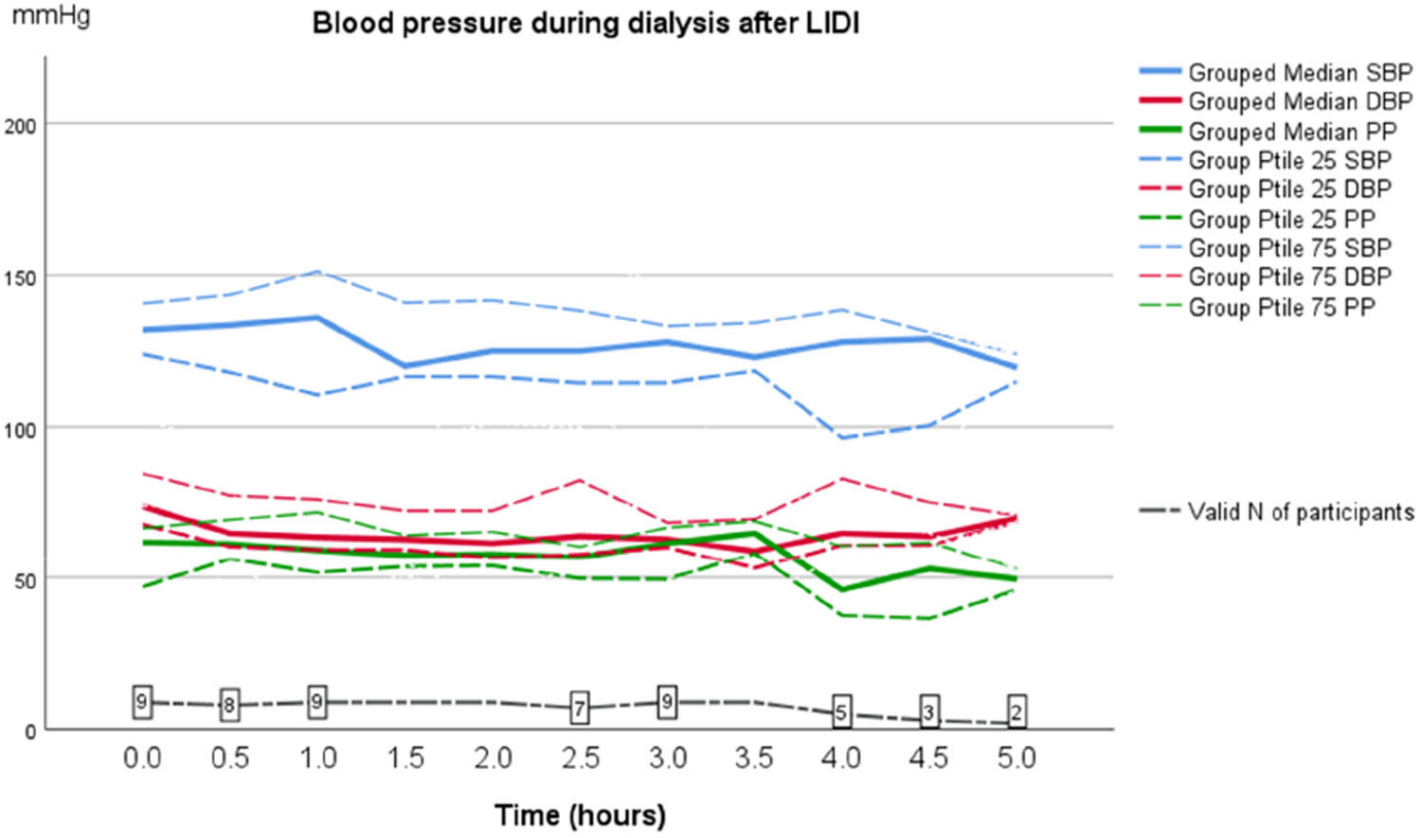
Ambulatory systolic and diastolic blood pressure (AMBP) measurements during the dialysis session after a long inter‐dialytic interval (LIDI). Data are median, 25th–75th percentiles and number of valid observations (*N*).

The pulse pressure too showed a trend to be higher after LIDI, but this difference did not reach statistical significance (data not shown). BP pre and post dialysis performed after the LIDI had a tendency (although not significant) to be higher than BP before and after dialysis after SIDI. Also, the difference between pre and post HD BP‐measurements differed numerically; after the SIDI median SBP decreased from 122 mmHg pre‐HD to 114 mmHg post‐HD, that is, 8 mmHg (*p* = 0.327), but after the LIDI the decrease was only a 3 mmHg, from median SBP 131 mmHg pre‐HD to 128 mmHg post‐HD (*p* = 0.362). Similarly, after the SIDI median DBP decreased from 69 mmHg pre‐HD to 58 mmHg post‐HD, that is, 11 mmHg (*p* = 0.092) and after the LIDI there was a 8 mmHg decrease in BP, from median SBP 76 mmHg pre‐HD to 68 mmHg post‐HD (*p* = 0.036) (Table 3). In addition, our observations showed that during the first 90 min of dialysis, BP dropped, regardless of after a SIDI or the LIDI as shown in Figures 5 and 6.

## 5 Discussion

The main finding of this pilot study was that the median systolic and diastolic intradialytic AMBP were significantly higher after a LIDI compared to after a SIDI. In addition, we found that during the first 90 min of dialysis treatment, BP dropped, whether after a short or long treatment break.

Our findings are in line with previous studies showing that pre‐dialytic, post‐dialytic and intradialytic BPs—in patients with three dialysis sessions per week—are higher after the longest inter‐dialytic interval (Kuipers et al. 2013). Also, a previous randomised controlled study showed that a smaller proportion of patients had hypertension, when measuring BP before dialysis treatment, if they had six dialysis sessions per week instead of three, thus when having a shorter inter‐dialytic interval (FHN Trial Group et al. 2010). Another randomised controlled study examined differences in pre‐dialytic and post‐dialytic BP associated with three alternative treatment schedules. This study too showed that both pre‐dialytic systolic and diastolic BP was lower with more frequent dialysis treatments and shorter inter‐dialytic intervals (Kotanko et al. 2015).

The importance of a joint, not isolated, interpretation of SBP before dialysis and intradialytic SBP change has been stressed previously (Zhang et al. 2020), but the differences in intradialytic AMBP, after different lengths of inter‐dialytic intervals are previously scarcely investigated. The reason for the observed difference in BP between dialysis treatments after different lengths of treatment interruptions was not investigated in this study. However, as intradialytic hypertension is linked to volume excess, one plausible explanation is greater fluid accumulation during longer treatment breaks, (Agarwal and Light 2010). This hypothesis was strengthened by the observed difference in intradialytic BP drop, only 3 mmHg after the LIDI verses 8 mmHg after the SIDI, suggesting that accumulated body fluid was not satisfactory removed during dialysis after the LIDI.

During night, BP is expected to drop. The absence of dipping, “non‐dipping”, which was observed in this study, is significantly more prevalent in patients with CKD and associated with increased risk for cardiovascular disease (Narita, Hoshide, and Kario 2022; Burnier and Damianaki 2023). During the second night in the inter‐dialytic interval AMBP did not drop, we even observed a tendency to increased BP. This increase was not statistically significant, however in line with previous findings, as in a cohort study, ambulatory aortic pressure was found to be higher on the third than the second day during inter‐dialytic intervals (Koutroumbas et al. 2015). In our study the inter‐dialytic observation time was limited to 44 h.

Only a small number of studies describing the BP variation over longer time periods have been published previously. A previous study described BP variability during dialysis treatment and the following 44 h until the next dialysis treatment, in patients receiving three haemodialysis treatments per week (Bikos et al. 2018), and van Buren et al. showed how systolic BP dropped in the first 24 h after dialysis treatment in patients who have intradialytic hypertension as opposed to those without dialytic hypertension who instead have a gradual rise in BP (Van Buren et al. 2011).

The drop in BP during the first 90 min of haemodialysis that our results suggest, could be due to ultrafiltering out the excess volume in the bloodstream and the volume surplus in the interstitium being mobilised more slowly into the bloodstream. Also, food intake during dialysis treatment can cause intradialytic hypotension (Agarwal and Georgianos 2018). In our study, the patients were served coffee during the dialysis treatment, but due to different starting times, not necessarily within the first 90 min of the treatment, hence not likely causing the drop in BP observed in the graph.

A major weakness of this pilot study, affecting the generalisability of the results, is the low number of participants and a possible selection bias, as recruiting was primarily based on the patients own motivation to participate in a research study. Also, there was a large individual variability among the patients, for example when it comes to time treated with dialysis, and only eight out of 11 participants had the same dialysis frequency. In another study examining BP in patients with CKD treated with haemodialysis, only patients with three dialysis treatments per week and 4 h dialysis treatments were included (Kuipers et al. 2013). On the other hand, this lack of harmonisation is natural for a pilot study. The measuring equipment has only been used in a few studies before, whereby reliable sources of validity could not be guaranteed. Finally, a large number of statistical analyses have been made without adjustment for “multiple testing” which, with a desired significance level of *p* < 0.05, means that false positive results (type 1 error) risk occurring.

One of the strengths of the study is that the patients are compared with themselves as their own controls. With the long measurement periods associated with ambulatory measurement, each variable will also be based on several measurements, which increases the reliability. Another strength of the study is the prospective design. All measurements and analyses in the study are done by the same person, thus reducing interobserver issues.

## 6 Implications for Clinical Practice

Because single BP measurements do not provide sufficient information for the management of high BP in patients with CKD Stage 5 and haemodialysis, ABPM is suggested to be used to complement standardised office BP readings (Cheung et al. 2021). This study shows that the measuring equipment could be used in connection with dialysis treatments and for longer periods than 24 h, without significant disturbances for the participants and with good data quality, and hence be used to potentially improve BP treatment in these patients.

## 7 Conclusion

In conclusion, this small pilot study measuring AMBP up to 52 h in 11 individuals with CKD treated with haemodialysis, showed that both systolic and diastolic BP differ between dialysis treatments following inter‐dialytic intervals of diverse length. This result needs to be confirmed in larger studies.

## Author Contributions

Jenny Stenberg participated in design and coordination, helped to draft manuscript, and read and approved the final manuscript. Oskar Sandberg participated in design, coordination, and data collection, helped to draft manuscript, read, and approved the final manuscript. Kerstin Marttala prticipated in design, coordination and data collection and approved the final manuscript. Maria K. Svensson conceived study, participated in design and coordination, helped to draft manuscript, and read and approved the final manuscript.

## Ethics Statement

The study complied with the Declaration of Helsinki and was approved by the Regional Ethics Committee in Uppsala (dnr 2021‐05658‐01).

## Consent

Written informed consent was obtained from all participants. Descriptive clinical characteristics were collected from electronic health records.

## Conflicts of Interest

The authors declare no conflicts of interest.

## Data Availability

Data are not publicly available but are available from the corresponding author upon reasonable request.
